# Factors associated with concerns about falling and activity restriction in older adults after hip fracture: a mixed-methods systematic review

**DOI:** 10.1007/s41999-024-00936-9

**Published:** 2024-02-28

**Authors:** Stefanny Guerra, Toby Ellmers, Ruqayyah Turabi, Magda Law, Aishwarya Chauhan, Rhian Milton-Cole, Emma Godfrey, Katie J. Sheehan

**Affiliations:** 1 Department of Population Health Sciences, School of Life Course and Population Sciences, Kings College London, London, UK; 2 Department of Brain Sciences, Faculty of Medicine, Imperial College London, London, UK; 3 Bone and Joint Health, Blizard Institute, Queen Mary University of London, London, UK

**Keywords:** Fragility fracture, Fear avoidance, Activity avoidance, Fear of falling, Balance confidence

## Abstract

**Purpose:**

To investigate factors contributing to concerns about falling and activity restriction in the community among older adults who had a hip fracture.

**Methods:**

A mixed method systematic review with a convergent segregated approach. We searched Medline, Embase, PsycInfo, PEDRo, CINAHL and the Cochrane library. Results were synthesised narratively considering physical, psychological, environmental, care, and social factors and presented in tables. Critical appraisal was completed in duplicate.

**Results:**

We included 19 studies (9 qualitative, 9 observational, 1 mixed methods) representing 1480 individuals and 23 factors related to concerns about falling and activity restriction. Physical factors included falls history, comorbidities, balance, strength, mobility and functionality. Psychological factors included anxiety and neuroticism scores, perceived confidence in/control over rehabilitation and abilities, and negative/positive affect about the orthopaedic trauma, pre-fracture abilities and future needs. Environmental factors included accessibility in the home, outdoors and with transport. Social and care factors related to the presence or absence of formal and informal networks, which reduced concerns and promoted activity by providing feedback, advice, encouragement, and practical support.

**Conclusion:**

These findings highlight that to improve concerns about falling and activity restriction after hip fracture, it is important to: improve physical and functional abilities; boost self-confidence; promote positive affect; involve relatives and carers; increase access to clinicians, and; enhance accessibility of the home, outdoors and transport. Most factors were reported on by a small number of studies of varying quality and require replication in future research.

**Supplementary Information:**

The online version contains supplementary material available at 10.1007/s41999-024-00936-9.

## Key summary points

**Aim:**

We conducted a mixed-method systematic review on factors related to concerns about falling and activity restriction among older adults after hip fracture.

**Findings:**

We identified several contributing factors related to the individual, formal care, environment, and social structure. We observed immutable factors that seem to worsen concerns and activity restriction, as well as modifiable factors that seem to help overcome fears and increase activity.

**Message:**

Management of concerns about falling and associated activity restriction after hip fracture needs a comprehensive approach that considers the various individual and external factors impacting fears and activity restriction in the community.

**Supplementary Information:**

The online version contains supplementary material available at 10.1007/s41999-024-00936-9.

## Introduction

Hip fractures are common in older adults, with UK hospitals admitting 75,000 older adults with hip fractures annually [1]. Whilst most individuals recover gait, balance, and both basic and instrumental activities of daily living (ADL) after a hip fracture [2, 3], a substantial proportion do not return to pre-fracture levels of physical function or activity [3–5]. Similarly, reductions in quality-of-life continue to occur in the months following hip fracture [4].

People with hip fractures have defined ‘recovery’ as returning to pre-fracture activities [6] which require being mobile in various life spaces. This in turn necessitates adequate physical capabilities and confidence about one’s balance [7, 8]. However, most patients report concerns about falling in the first three months after a hip fracture [9]. Concerns about falling refer to “a lasting feeling of dread and apprehension about situations that are believed to threaten or challenge balance” [10]. These often lead to the restriction and/or avoidance of physical and social activities [10–12] which can then trigger a downward spiral of deconditioning, increasing physical frailty, falls, and social isolation [10, 13–15].

Trajectories of concerns about falling following hip fracture are complex. Evidence suggests the initial increase in concerns about falling at 4 and 8 weeks post fracture typically reduces by 3 months [9, 16, 17], although some individuals have concerns that persist for at least 6 months [18, 19] and up to 13 months after hip fracture [9]. A study investigating physical activity trajectories in the English Longitudinal Study of Ageing reported that older age and greater frailty were associated with lower physical activity participation after hip fracture [20]. To our knowledge, no review to date has investigated factors associated with activity restriction after hip fracture.

Several studies have identified factors of concern about falling after hip fracture surgery, such as pre-fracture activity, falls history, living alone, taking over four medications, post-fracture mobility and difficulties with basic ADL [18, 21]. A recent review reported that concerns about falls after hip fracture are consistently observed among people with poorer pre-injury physical function [9]. Concerns about falling after hip fracture have been associated with further falls [21], institutionalisation [21], low mood [18], lower functional abilities [18] and poorer outdoor mobility recovery [21]. Many studies exploring this topic focused on quantitative data collected at varied time points (e.g., from hospitalization up to seven years after discharge), and have not included qualitative studies.

The current review aims to synthesise the quantitative and qualitative evidence on factors of concerns about falling and activity restriction after hip fracture surgery in the community. We decided to focus on factors captured after discharge to better identify those for whom concerns about falling may be more severe and longer lasting, potentially leading to poorer outcomes. This review will enable a better understanding of who is at risk of developing concerns about falling following a hip fracture for future targeted interventions.

## Methods

### Design

We adopted a mixed methods systematic review design to identify factors that may be perceived by individuals in qualitative studies and/or quantified in observational studies. These studies are likely to identify different aspects of concerns about falling after hip fracture, so we followed a convergent segregated approach [22] where both quantitative and qualitative study designs are considered of equal importance and synthesised simultaneously and separately [22]. We registered the review on the International Prospective Register of Systematic Reviews (PROSPERO ID: CRD42022338881) and reported it in adherence with the updated referred Reporting Items for Systematic Reviews and Meta-Analyse (PRISMA) statement [23].

### Eligibility criteria

We included observational and qualitative studies collecting data in the community, and targeting older adults (mean or median age of 50 years or older) who had previously undergone surgery for a non-pathological hip fracture. We included studies investigating the relationship between the presence/absence of any prognostic factors and ‘fear of falling’, ‘falls-related self-efficacy’, ‘activity avoidance’, ‘activity restriction’, and/or ‘balance confidence’ across care settings. We used ‘fear of falling’ as the umbrella term for our searches, as this term is most common in the literature prior to the publication of the recent World Falls Guidelines where the term ‘concerns about falling’ was instead recommended [24]. Lastly, we excluded intervention studies, studies limited to inpatient settings, conference proceedings, editorials, commentaries, case-studies, case-series, and non-English language studies.

### Search strategy and screening

We searched Medline, Embase, PsycInfo, PEDro, CINAHL and the Cochrane library from database inception to the week commencing June 17th, 2022. We developed a search strategy using a combination of free text words and controlled search terms for hip fracture and fear of falling and/or activity restriction (Supplementary Appendix I). Hip fracture [25, 26] fear of falling [27], prognostic factors [28] and qualitative [29] terms were adapted from published search strategies. Title, abstract and full-text screening were completed in duplicate (ML, AC and SG) using the platform Covidence [30]. Reference lists of relevant studies and reviews were hand-searched [31]. Disagreements were resolved by consensus.

### Data extraction, synthesis, and appraisal

Following a convergent segregated approach, we extracted, synthesised and integrated observational and qualitative papers simultaneously and separately [22]. Primary mixed methods studies were classified as individual observational and qualitative studies.

For observational studies, we extracted authors’ names, publication year, country, study design, eligibility criteria, participant description, sample size, analysis approach, prognostic factors (definition and timing of measurement), outcomes (definition and timing of measurement), effect estimates and measure of dispersion, and main narrative results, in duplicate (ML, AC, SG). Observational studies were heterogeneous with respect to associations explored, outcomes, and reporting of results and not amenable to meta-analysis [32]. Therefore, we synthesised observational studies narratively [33] by: (i) a preliminary synthesis focused on effects’ size and direction, and of any patterns arising; (ii) exploration of the relationships within and between reports through tabulation of studies’ results and characteristics; and (iii) assessment of the robustness of studies through quality appraisal in duplicate (SG, RMC) [33]. We followed the Quality in Prognostic Studies (QUIPS) tool to assess the risk of bias in study confounders, participation and attrition, prognostic and outcome measurements, and statistical analysis and reporting [34]. We assigned each study a ‘low’, ‘moderate’, or ‘high’ risk of bias in each of these fields.

For qualitative studies, we captured descriptive data using a standard data extraction form [35]. One author (SG) used a selective approach [36] to extract relevant findings data on factors perceived to influence concerns about falling and/or activity restriction. ‘Findings data’ was considered from text and tables published in the ‘Results’ section [35]. Subsequently, SG followed a narrative synthesis approach [37] using NVivo (version 12). To calibrate data extraction, a subset (*n* = 4) of studies were extracted and analysed independently by RT. On comparison, similar themes were yielded. Both reviewers followed the following steps: (i) Inductive free coding, line- by-line, of data (disregarding the research question) (ii) Grouping free codes into descriptive themes, (iii) Deriving analytical themes by inferring concerns about falling and/or activity restriction factors from descriptive themes, and (iv) assessment of the robustness of studies through quality appraisal in duplicate (SG, RT) using the Critical Appraisal Skills Programme tool (CASP) for qualitative studies [38]. With the CASP checklist, we answered 10 questions regarding each study aims, methodology, design, recruitment, collection, reflexivity, ethical issues, data analysis, findings, and implications discussed. We assigned each study a ‘Yes’, ‘No’, or ‘Uncertain’[38].

**Fig. 1:**
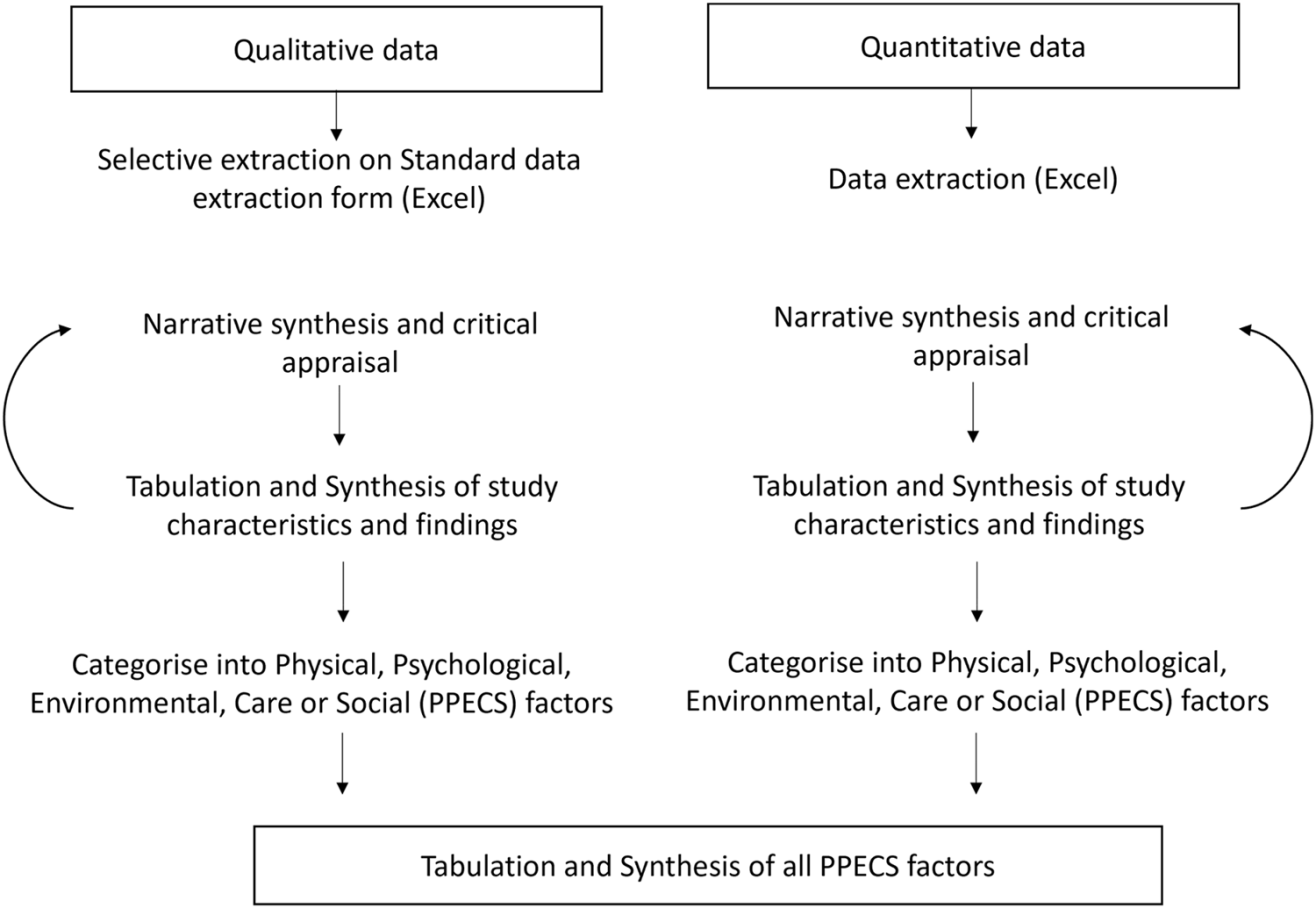
Extraction, synthesis and appraisal of qualitative and observational studies following a convergent segregated approach

Factors identified in descriptive themes (qualitative studies) and associations (observational studies) were then classified by SG as Physical, Psychological, Environmental, Care, or Social factors (Fig. 1). We did not transform data (i.e., from qualitative to quantitative), but instead analysed and provided a synthesis of both types of evidence [22].

## Results

### Search

**Fig. 2:**
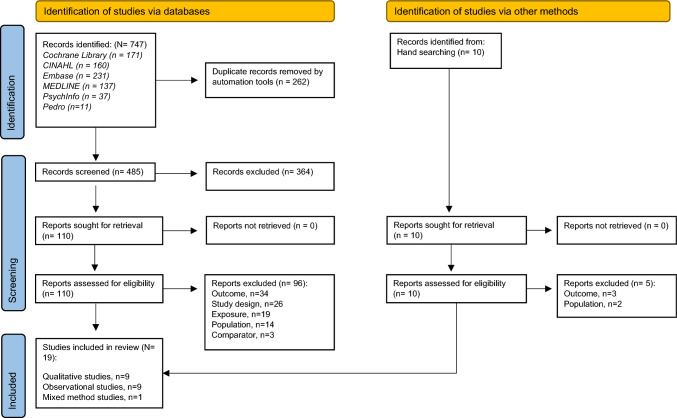
PRISMA flow diagram

We identified 485 papers following de-duplication and 10 from hand searches of reference lists (Fig. 2). In total 375 were excluded on title and abstract screening, and a further 101 were excluded following full-text screening. We included 19 papers in the review, representing 1 mixed method [39], 9 qualitative [8, 40–47] and 9 observational [19, 48–56] studies.

### Study characteristics

#### Observational

We included nine observational studies and one mixed-method study of cross-sectional [39, 52–54], prospective [19, 48–50, 55] and retrospective [51] designs, conducted in Europe [39, 48, 51, 53, 54], North America [49], Japan [50, 52] and Australia [19, 55] (Supplementary Appendix II). The studies reflected analyses from 1330 older adults after hip fracture (sample size range 33 [39] to 263 [49]). Participants were mostly women (68% [51] to 100% [52]), and the mean age ranged from 64 [52] to 83 [48] years old.

#### Qualitative

We included nine qualitative studies and one mixed-method study which were conducted in Europe [39–44, 46, 47] Brazil [45] and Australia [8] (Supplementary Appendix II). The research was of inductive [41] phenomenological [8, 45–47], grounded theory [8, 43, 44], or unspecified [39, 40, 42, 46] design. The studies reflected 150 older adults after hip fracture (sample size range from 4 [39] to 31 [40]). Most participants were women (from 64.5 [42] to 88% [41]), and the mean age ranged from 74 [46] to 85 [40] years. One study interviewed both carers and patients [42]. Four interviewed participants multiple times [40–42, 46]. Two publications [43, 44] analysed the same dataset.

### Outcome

#### Observational

Outcomes were measured with the Activities-specific Balance Confidence scale (ABC) [51, 53, 54, 57], the Falls Efficacy Scale (FES) questionnaire [50, 58], the FES-International (FES-I) [48, 59], the short FES-I [19, 49], a modified FES [52], both the ABC and FES [55], or both the FES-I and the modified Survey of Activities and Fear of Falling in the Elderly [39]. Outcomes were assessed at hospital admission [48, 49], 2 to 6 weeks post-fracture [19, 49, 50, 53], 3 to 6 months post-fracture [19, 39, 48, 50, 53, 55], and up to 7.5 years post-fracture [51, 53, 54]. One study did not specify when assessments in the community took place [52].

#### Qualitative

Studies explored concerns about falling and/or activity restriction through a variety of research questions, such as the overall impact of a hip fracture [40–42, 47]; or how mobility [8], adaptations [41], concerns [43, 44], sedentary behaviours [45, 46], and fear of falling [39] changed post-fracture. Data was collected during hospital admission [8, 40], 2 to 12 weeks post-fracture [8, 40, 42–44, 46], 3 to 6 months post-fracture [39–42, 46], and over 6 to 24 months post-fracture [40, 45, 47].

### Critical appraisal

#### Observational

Six studies [39, 48–50, 52, 55] had a high risk of bias in at least one of the ten QUIPS domains. Four of these [39, 48, 50, 55] were on confounders and two were on study attrition [49, 55]. Moderate risk of bias was determined for all studies in at least one domain. Most (*n* = 9) [39, 48–53, 55, 56] were on the prognostic factor measurement, five [49, 52–54, 56] on study confounding, and four [39, 48, 50, 55] on statistical analysis and reporting.

#### Qualitative

Almost all studies clearly stated research aims that were adequately answered by qualitative studies, appropriately recruited participants, and collected data (CASP item 1, 2, 4, 5). Most provided detailed information on the analysis steps, provided representative quotes, and discussed findings contributions and usability/future steps (item 8, 9, 10). Over half of the studies (*n* = 6) failed to critically examine researchers’ relationships with participants (item 6) [8, 40, 41, 43–45]. Four failed to justify their research design (item 3) [8, 40, 41, 44] (Supplementary Appendix III).

### Factors

Tables 1 and 2 show study findings related to concerns about falling and activity restriction. Table 3 presents a summary of all factors classified as physical, psychological, environmental, social or care factors. Evidence from both study designs is summarised below.

**Table 1:** Findings of observational studies

References	Sample size	Factors (measure)	Timing measure	Outcome (measure)	Timing measure	Findings
Benzinger et al. [48]	51	Age (self-reported) ADL functioning (Barthel Index) Short physical performance battery Pain (WOMAC*) Cognition (SOMC—Short Orientation Memory Concentration test) Depressive symptoms (GDS)	Within 2 days of admission to a rehabilitation unit 4 month follow-up	FES-I, German version Primary outcome	Within 2 days of admission to rehabilitation unit 4 month follow-up	At baseline, FES-I correlated with: age (*r* = 0.56, *p* < 0.001), baseline ADL performance (*r* = –0.31, *p* = 0.028), pain (*r* = 0.43, *p* = 0.002), At 4 months follow up, FES-I correlated with: age (*r* = 0.48, *p* < 0.001), depressive symptoms (*r* = 0.40, *p* = 0.004), ADL performance at follow-up (*r* = –0.59, *p* < 0.001) Pain at follow up (*r* = 0.16, *p* > 0.005)
Bower et al. [49]	263	Age, gender, pain (self-reported) Comorbidities pre-fracture (CIRSG) Neuroticism (IPIP) Functional ability pre-fracture (FRS) Positive and negative affect (PANAS) Cognition (SBT) Depression (MADRS) Social Support (DSSI) Negative affect (PANAS) Pain (numerical scale)	1 week post hip fracture	Short FES-I Primary outcome	1-, 4- and 12-weeks post fracture	Increasing FOF—Multivariate models OR (95% CI): CIRSG = 1.70 (not provided), *p* = 0.012 IPIP Neuroticism = 1.72 (not provided), p = 0.013 Decreasing FOF—Multivariate models OR (95% CI): FRS = 0.67 (not provided), *p* = 0.012 IPIP Neuroticism = 1.68 (not provided), *p* = 0.005
Goto et al. [50]	72	Pain intensity [5-point Verbal Rating Scale(VRS)]	2, 4, 8 weeks after surgery	FES Secondary outcome	2 and 4 weeks after surgery	FES mean (SD) 2 weeks after surgery, Light pain group = 24 (6.5) Severe pain group = 22.2 (7.8), *p* = 0.34 FES mean (SD) 4 weeks after surgery Light pain = 26.7 (5.6) vs Severe pain = 23.6 (6.8); *p* = 0.05
Jellesmark et al. [39]	33	Avoidance of activities due to FOF (mSAFEE) Functional ability—ADL (FRS) Functional ability—mobility (NMS)	3 months post-discharge	FES-I Primary outcome mSAFEE Covariate	3 months post-discharge	FES-I correlated (r) with MSAFFE = 0.83 FRS = − 0.78 NMS = − 0.67 MSAFFE correlated (*r*) with: FRS = − 0.80 NMS = − 0.74 Higher vs lower FES-I scores (median [IQR]) comparisons: mSAFFE: 33 (22–43) vs 19 (16,0–20.5), *p* < 0.0001 FRS: 71 (55.0–81.0) vs 90.5 (84.5–95.5), *p* < 0.0001 NMS: 4 (3–6) vs 7 (7–9), *p* < 0.0001
Kulmala et al. [51]	79	Fall status (self-reported), including: falls, recurrent falls, indoor and outdoor falls	6 months to 7 years post-fracture	ABC Primary outcome	6 months to 7 years post-fracture	ABC mean (SD) differences Recurrent falls = 68 (51) vs No recurrent falls = 97 (31), *p* = 0.023 Falls indoors (72 ± 35) vs No indoor falls (100 ± 32), *p* = 0.004 ABC Unadjusted OR (95% CI) Recurrent falls: 0.97 (0.95–1.00), *p* = 0.032 Indoor falls: 0.98 (0.96–1.00), *p* = 0.037 ABC Adjusted OR (95% CI) Indoor falls 0.98 (0.96–1.00), *p* = 0.037
Nagai et al. [52]	214	History of falling (self-reported questionnaire) Time since THA, bilateral THA, use of multiple medications (study assessment) Ambulatory status/walking capacity (self-reported question ‘how long can you walk without stopping’) Functionality and pain (OHS) Anxiety (PSWQ)	Post-discharge, unspecified time	FES (modified by authors to exclude 1 item and include 3) Primary outcome	Post-discharge, unspecified time	FES correlated with OHS: *β* = 0.18; *p* = 0.008 History of falling in the past year: *β* = 0.17; *p* = 0.010 Walking capacity: *β* = 0.14; *p* = 0.034), Total PSWQ score: *β* = 0.15; *p* = 0.020 Age: *β* = 0.14; *p* = 0.036
Portegijis et al. [53]	130	Age, sex, time since fracture, type of surgery, comorbidities, knee extension strength (study assessment) Walking speed (TUG) TUG test time Self-reported mobility (questionnaire) Functional balance (BBS) Physical activity level (self-reported scale by Grimby*)	6 weeks to 7.5 years post-fracture	ABC, modified Finnish version Primary outcome	6 weeks to 7.5 years post-fracture	ABC correlated with (spearman *r*, pearson *p*) Age: *r* = 0.37, *p* < 001 Muscle strength, *r* = 0.40, *p* < 001 Walking speed, *r* = 0.51; *p* < 001 TUG test time, *p* = − 0.56.; *p* < 001 BBS score, *p* = 0.72; *p* < 001 Physical activity, *p* = 0.47, *p* < 001 Number of diseases, *p* = − 0.29, *p* < 001 Ability to walk outdoors. *p* = − 0.54; *p* < 001 Stair climbing ability, *p* = − 0.57; *p* < 001
Salpakoski et al. [54]	78	Physical activity (interview, YPAS) Musculoskeletal Pain in Lower Body (Visual Analog Scale)	8 months to 7.5 years post-fracture	ABC Covariate	8 months to 7.5 years post-fracture	ABC mean (SD) differences Physical inactive = 80.4 (35.1), vs Physically active = 102.2 (31.1), *p* = 0.007 Low to No-Pain = 103.0 (30.1), vs Severe Pain = 82.9 (36.1); *p* = 0.01
Tu et al. [19]	34	Physical activity levels (Human Activity Profile—Adjusted Activity Score and Frenchay Activity Index) Mobility (walking speed, 6‐ or 10‐m walk test) Balance (Timed Up‐and‐Go test and Step Test) Falls history post-discharge (self-reported) Pain (analogue scale) Self-efficacy (SES)	At discharge	Short FES-I Primary outcome	2 weeks, 3 and 6 months after discharge	Short FES-I, mean 95% CI 2 weeks to 3 months = -2.1 (3.6 to − 0.6), *p* < 0.005 2 weeks to 6 months = − 3.0 (− 4.6 to − 1.5), *p* < 0.001 Non fallers 2 weeks to 3 months = –1.9 (–3.7 to –0.1), *p* = 0.041 2 weeks to 6 months = –2.4 (–4.4 to –0.5), *p* = 0.014 Fallers 2 weeks to 3 months = –2.5 (–5.0 to 0.0), *p* = 0.046 2 weeks to 6 months = –4.1 (–7.1 to –1.1), *p* = 0.007
Whitehead et al. [55]	183	Gait speed (10 m walk) History of falling post-fracture (self-reported)	4 months post-discharge	ABC and FES Covariates	4 months post-discharge	FES mean (SD) differences Fallers = 61.7 (22.6) vs No fallers = 73.5 (26.2), *p* < 0.05 Slow walking speed = 71.3 (22.9) vs Normal walking speed = 78.6 (33.8), *p* < 0.05 ABC mean (SD) differences Fallers = 3.4 (20.1) vs No fallers = 53.5 (23.0), *p* < 0.05 Slow walking speed = 45.6 (21.0) vs Normal walking speed = 75.5 (16.6), *p* < 0.001 FES correlated with (*r*): ADL = 0.589, *p* < 0.001 LHS = 0.617, *p* < 0.001 BBS = 0.548, *p* < 0.001 ABC correlated with (*r*): Gait speed = 0.650, *p* < 0.001 ADL = 0.667, *p* < 0.001 LHS = 0.795, *p* < 0.001 BBS = 0.772, *p* < 0.001

Activities-Specific Balance Confidence scale (ABC). The Cumulative Illness Rating Scale for Geriatrics (CIRSG). Duke Social Support Index (DSSI). Functional ambulation categories (FAC). Falls Efficacy Scale (FES). Fear of falling (FoF). The Functional Recovery Score (FRS). Geriatric Depression Scale 8-item version (GD S8). Hospital Anxiety and Depression Scale (HADS-A). Mini International Personality Item Pool (IPIP). Montgomery Åsberg Depression Rating Scale (MADRS). Modified Barthel Index (MBI). The modified survey of activities and FOF in the elderly (mSAFFE). New Mobility Score (NMS). Positive and Negative Affect Schedule (PANAS). The Japanese version of the Penn State Worry Questionnaire (PSWQ). Oxford Hip Score (OHS). The modified Timed-Up-and-Go (TUG). Short Falls Efficacy Scale International (Short FES-I). Self-Efficacy Scale (SES). Short Blessed Test (SBT). Western Ontario and MacMaster Universities osteoarthritis index (WOMAX). Yale Physical Activity Survey (YPAS)

**Table 2:** Findings of qualitative studies

References	Sample size	Data collection (duration), timing, location	Data analysis approach	Data extracts examples
Abrahamsen et al. [40]	12	Semi-structured interviews (lasted 20–28 min) During admission, face-to-face (*n* = 12) 2–6 weeks post-discharge, by telephone (*n* = 6) 5–6 months post-discharge, by telephone (*n* = 4) 12 months post-discharge, by telephone (*n* = 4)	Abductive reasoning	1.1 Theme: Briefly after the fall: during admission: Fear of falling was a great concern for many, especially when going to the toilet at night (ID 7) 1.2 Theme: Briefly after discharge: in the home Five of the six patients interviewed after discharge reported they had not left their home since discharge as their mobility was reduced and they still worried about falling: “My leg gets tired in the middle of the day […] and the stairs prevent me from getting out” (ID 8) 1.3 Theme: Five to six months after the fracture: continuous improvement Those who had previously been able to go for walks outside, manage their shopping and gardening and enjoy social events regained their pre-fracture abilities by using a walker. Yet using public transport or biking remained out of reach, or they did not dare it anymore as they feared falling again. As one said, “getting off the bike makes me nervous of falling again” (ID 11) 1.4 Theme: Twelve months after the fracture: taking stock. One year later most of the patients had regained their previous level of physical functioning and were able to perform almost all usual activities of everyday life except for some outdoor activities such as taking the bus or biking because they were “afraid of falling” (ID 7)
Gesar et al. [41]	25	Semi-structured interview (lasting 38–63 min) 4 months after hip fracture, at participants home (*n* = 24) and at a café (*n* = 1)	Inductive content analysis	2.1 Uncertainty in physical activities has psychological effects The long-lasting insecurity about walking properly and the fear of falling again, acting in opposition to the motivation to remain independent, was a real challenge for some participants. The need to adapt to their impaired mobility was described as having an inhibitory effect. They had to adjust their daily activities to a slower rate due to their insecurity regarding their physical abilities. This resulted in the participants becoming more hesitant in taking initiative to perform physical activities. As a consequence of this immobility, everyday life had become isolated. “…Now, I am not as active as I used to be. I am now much more afraid of falling again. I am at zero now and have to push myself. I do not walk outdoors like I used to do. No spontaneous activities because everything has to be carefully prepared …” (Woman, 89 years) 2.2 To generate a strong driving force and determination is the basis for recovery after an operation Some participants had adjusted their daily activities to a slower rate. They described that the essentials for regaining self-esteem and self-confidence included managing everyday chores by themselves at a time chosen by them
Griffiths et al. [42]	31	Semi-structured interviews (20–90 min) 4 weeks and 4 months post fracture, at their current residence or in hospital	Thematic analysis, and cross-case analysis	4.1 Fear of Falling The experience of the fracture left a few participants with a fear of falling and sustaining a further fracture “I think it frightened him more than anything else. He’s frightened he’ll fall over again and do it again, that bothers him more than anything else. Because now when he stands up at all to try and walk he’s frightened he’s going to fall over and the same thing will happen all over again.” (Carer of participant 11, male, age 84, 7 weeks post operation) “I’ve got to watch what I’m doing. If I catch my foot on [paving stone], I can go over again.” (Participant 12, male, age 78, 16 weeks post operation) The fear of falling was sometimes expressed by a family member. When talking about his frustration at not being able to work in the garden, participant 6 added: “All the rain has made it very slippery, and [wife] says, “No way do you go out there.”” (Participant 12, male, age 78, 6 weeks post-operation). This emphasises the value given to mobility without falls or fear of falls by interviewees 4.2 Recovery through adaptation For some, their own or their carer’s fear of further falls limited their mobility or at least limited how far they tested their ability to walk. Poor weather conditions exacerbated this fear, but adaptations to the environment such as walking aids or handrails lessened the fear
McMillan et al. [43]	19	Semi-structured interviews (lasting 34–70 min) 2 and 12 weeks after discharge, face-to-face in participants’ homes	constant comparative method	5.1 The second stage: keeping afloat As people attempted to take control by balancing the help available to them, they simultaneously took control by balancing risk. The main strategies that people used were protective guarding and following orders. In the post-discharge context, people needed to learn to balance risk to make progress, and older people were aware of the potential dangers as they tried to rebuild their damaged confidence: ‘I mean on the one hand, you’ve got this fear of falling and on the other, you are trying to do things.’ (Participant 6) ‘Following orders’ was about being given strategies to balance risk by healthcare professionals, rather than people having to devise strategies for themselves. Although others were initially in control, people demonstrated their determination to recover by following orders and demonstrated understanding of the negative consequences of not following orders whilst keeping afloat: ‘you are doomed.’ Following orders was enhanced when coupled with explanation: ‘I watch myself very carefully, I was told when I stand up [demonstrates] to stand still for quite a bit, that’s to let the blood pressure come back to normal because blood pressure is different when you are standing to what it is when you are sitting, so I wait for a wee while before I finally take the first step.’ (Participant 14)
McMillan et al. [38]	19	Semi-structured interviews (lasting 34–70 min) 2 and 12 weeks after discharge, face-to-face in participants’ homes	constant comparative method	6.1 Balancing risk by following orders This may appear to be a non-conformist dimension of following orders; however, it reflected the older person’s fear of falling. This fear drove their attempts to contain risk: “Before [hip fracture] I could walk quicker. It’s worse here [back at home], trying to get to the toilet, sometimes I can manage and other times it is running out of me. I didn’t take my water tablet this morning. When the district nurse came in to see about my legs, she says, why not, I says because it is running out of me, well it doesn’t matter she says, you’ve still got to take your water tablet.” (Participant 4) Therefore, during the early stages of recovery, some people perceived that following some advice meant they would be at risk of having a further fall. They chose to balance risk by not following these orders. As people made progress, their reasons for not following advice changed, as they then started to perceive some advice as threatening to their independence, rather than as a threat to experiencing a further fall 6.2 The significance of being informed Therefore, balancing risk safely was a consequence of being provided with adequate information. Being informed is related to receiving information, feedback, advice or reassurance from healthcare professionals regarding progress. Older people had to rebuild their damaged confidence, unsure of their abilities and needed encouragement to increase their self-efficacy. Older people valued being included in discussions about their progress and future abilities. Being able to recognise progress and acknowledge achievements was important to them and contributed to enhancing their self-efficacy
Moraes et al. [45]	11	Face-to-face interview (average duration not reported) Between 6 and 24 months after a hip fracture surgery, based on participant’s preference	Discourse analysis Phenomenological approach	7.1 Barrier 3: fear of falling The experience of the fracture left some participants afraid of falling and led to a decrease in self-confidence, keeping participants more time seated and less active based on the fact that the primary concern was to avoid a new fall episode. Ruby: “I’m afraid, I’m afraid of falling and hurting myself again [...] Because I’ve had three falls.” Angelita: “In the street you feel insecure, and a little afraid of falling, because I don’t want to fall again, do I? [...] I’m afraid of falling.” 7.2 Facilitator 2: having a caregiver For the interviewees, having a support network, whether formal or informal, was indicated as a way of overcoming dependency to move around and, especially, to walk safely, resulting in better mobility and more active behavior
Jellesmark et al. [39]	4	In-depth interview using Semi-structured interviews guide (lasted 60–90 min) 5–6 months post hospital discharge, location not stated	Systematic text condensation	8.1 Fear of falling The main concerns were injury, pain and inability to rise after falling. According to the informants, a walker reduced FOF and was necessary at least when walking outside. It was a source of security, but also a nuisance and barrier to moving freely. They felt stigmatized when using a walker. Another complaint was the inability to use public transportation because the walker was unmanageable in a bus or a car. The radius of mobility was reduced and the informants required assistance. Kim explained: ‘I´m unable to visit my husband’s grave. It´s impractical… my brother-in-law picked me up, and we drove out there together. But he can´t do this too often… If I took the train to [city], it´s still far and there aren´t any busses that go to the cemetery’ FOF could be reduced by taking precautions such as using a shower bench or an emergency call system. Other precautions reduced their activity level. A chair or sofa became a place of safety. ‘… yes, it is important… I can´t do… it [fear of falling] makes me lazy, I believe. I prefer to sit in this soft chair (pats the backrest of a chair) or to lie there (points to the sofa), so, this is what I need… to be drawn out of the house to another place’… (Kim) Limited mobility was in part due to FOF, but also to pain, muscle weakness, impaired balance and lack of energy. Even 5 or 6 months after the hip fracture the informants had not regained the expected level of mobility. Three informants were aware of the importance of keeping fit and going outside for fresh air, but they were more limited than before and this led to frustration. Kate feared confinement to her home: ‘… Then I thought, I must try, if I can walk by myself, and I did all right last week, but suddenly I got… pain in the knee, and then, you know, I get afraid of not being able to walk back to my home. I´m not sure if I should continue or if I should just walk indoors, but fresh air is good for me’
Rasmussen et al. [46]	9	Semi-structured interviews: 2 weeks post-discharge, face-to-face (participants homes, day centre, rehabilitation centre) 6 months post-discharge, face-to-face (participants homes, nursing room)	Phenomenological-hermeneutic	11. 1 Inner driving forces A sense of belonging was a driving force related to sharing and being in this together with other people, having a feeling of being connected with and still belonging to the world. When the body was not able to carry out meaningful everyday life projects, participants were reaching for a normalization of their life. Sharing practical tasks and problems, newspapers, and celebrations brought joy, contentment, and the value of progress. Through long relationships, family members knew their preferences and routines, and Lene explained how they took care of “small things you normally do not count, but just do. But in my present situation, they are big things.” Particularly for participants recently discharged and isolated in the home, visits were energizing; and for Joan, friends visiting made her feel that “they haven’t forgotten you”. […] A sense of identity was a driving force related to being able to do things independently and feeling dignified. Participants were aiming for homecoming in the sense of being in touch with personal capacities and values. Being persistent, creative, positive, vigilant, and thoughtful supported a sense of being, and experiences of progress maintained hope and self-confidence. Responsibility was a matter of finding solutions to problems and having duties. Karen had managed baking cookies for my second visit: “I tell myself, you HAVE to try, and then when a full baking sheet is ready, I go and sit down for a while. Then back to the oven again. It can take a long time but I have nothing but time”
Taylor et al. [8]	24	Semi-structured interviews (20 and 40 min) average weeks post-surgery (SD) = 3.9 (2.4), and 12.2 (4.9) weeks, at participants’ convenient location	Phenomenological theoretical framework, and grounded theory method	9.1 Walking function at home after hip fracture. A key theme emerged of reduced walking and mobility both at home and in the community. All participants were either not walking outside or walking much less than they used to: “I wouldn’t even attempt walking outside... I feel a bit like a prisoner.” (Olive) Participation in community activities was much reduced. Most participants said that they had either not gone shopping since the fracture, or they had only been shopping once or twice with the assistance of family members. Participants also mentioned that they had not returned to previous activities such as going to church, study group, book club, or golf; and others said that they were reliant on a family member to get them to medical appointments. Participants also reported reduced levels of mobility around home. Although it was an effort, all were walking in the house. However, most participants were either doing much less or no housework: I do very little (housework). Very, very little. (Rose) The factors associated with reduced walking at home and in the community were themed as psychological, physical, social/environmental. Psychological factors emerged as a strong theme, most commonly described as a lack of confidence and a fear of falling: “I’ve never had a fall before. 86 years and I didn’t have a fall, so now I’m a bit frightened.” (Thelma)
Ziden et al. [47]	15	Semi-structured interviews [lasted for an average 27 min (range15–39 min)] 12 months post-discharge, participants’ home	Phenomenographic method	10.1 Isolated life, restricted activity and fewer social contacts All interviewees expressed that the hip fracture had caused remaining negative consequences for their daily life. This was expressed as either psychological reactions, such as being more uncertain and afraid, or more physical restrictions, such as being more limited to move and to do the things they used to do before the injury. They described that this had meant activity restriction, fewer social contacts and an isolated life, compared to before the fracture 10.2 More insecure and afraid Within this sub-category the subjects expressed new or increased feelings of insecurity as a consequence of the injury. This included fear of falling again and perhaps getting a new fracture, of spoiling the surgery or having to go through a re-surgery, or of becoming a burden to relatives or friends. Some interviewees described the shock after the injury in words such as ‘everything became a mess’, and that the injury had brought feelings of insecurity and distress “You think, why didn’t I put the light on when I got up [at night]?... So, I’m very careful now, almost excessively so.... I’m careful when I’m out walking... Then I take it really easy! Look down at the ground... Now I’m afraid that it will happen again. And [that I will] break something else.” (subject 9)

*FoF* Fear of falling

**Table 3:**
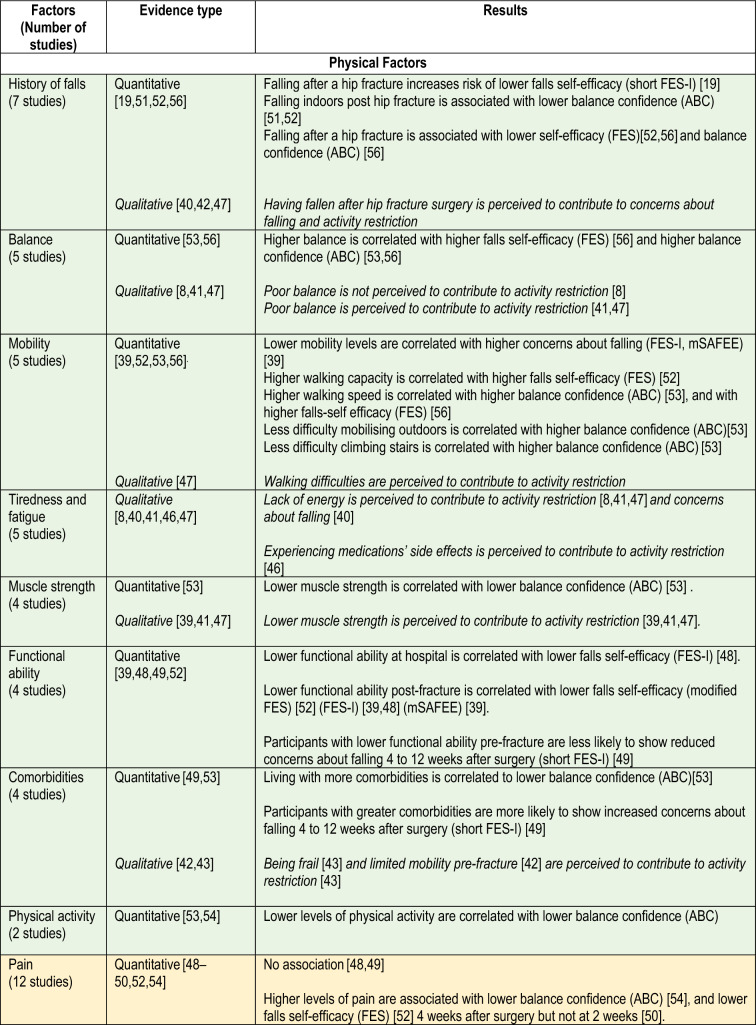

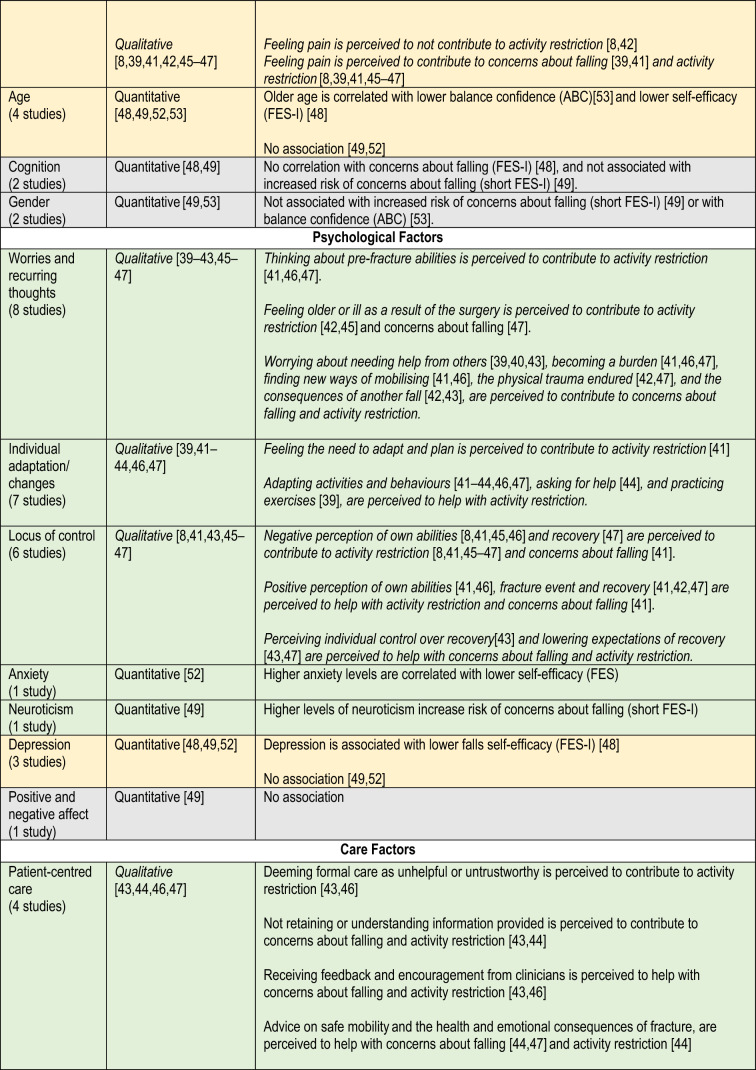

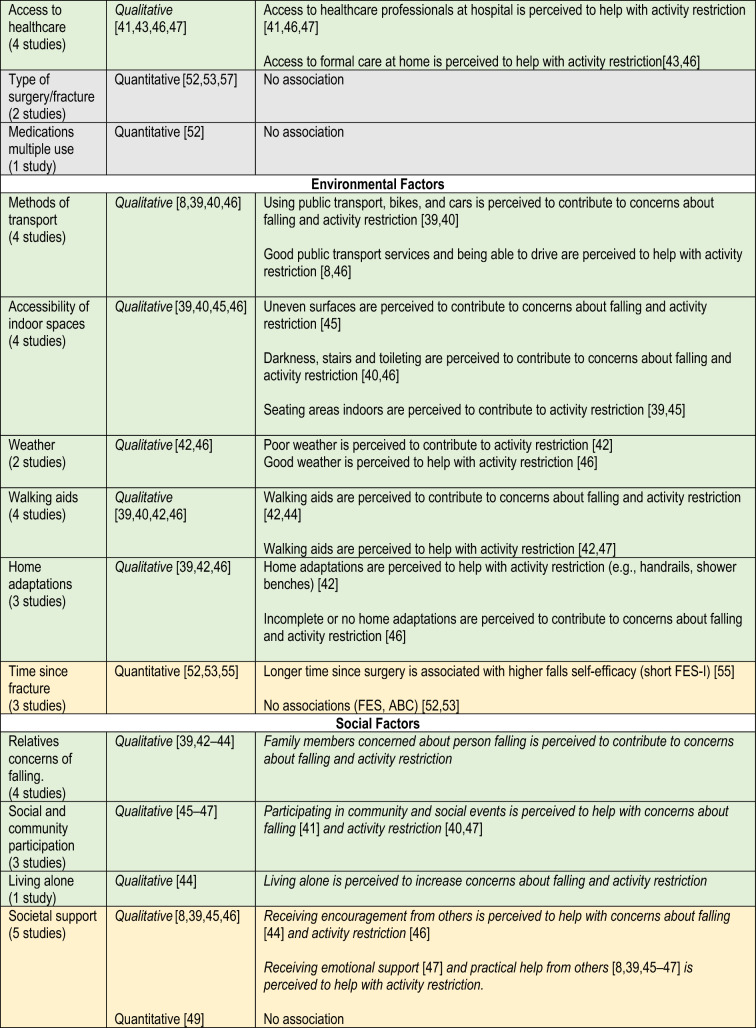
Physical, psychological, care, environmental, and social factors identified

Green = association found in observational studies, and/or factor described in qualitative studies. Yellow = conflicting/mixed evidence from observational and/or qualitative studies. Grey = association not found in observational and/or qualitative studies

#### Factors of concerns about falling and activity restriction

Observational studies reported more concerns about falls and activity restriction among participants who fell after their surgery, lived with comorbidities or had poorer mobility and functionality post-fracture. In qualitative studies, participants attributed low mobility pre-fracture, having fallen again after surgery, fatigue and lower strength, as influencing factors for concerns and reduced activity (*Physical*). One primary source summarised these concerns:

*“Four of the interviewees had experienced a new fall after discharge, which they thought had added to their fear of falling. The deteriorated ability to move and walk had made them lose power and strength, mentally as well as physically, and they experienced that they had become more tired than before.”*[47]

In observational studies, concerns about falls were seen among participants with higher scores on anxiety and neuroticism scales. Authors of qualitative studies noted that concerns about falling and activity restriction co-existed with diminished confidence in recovery and own abilities to engage in physical therapy and overcome challenges. Participants who acknowledged concerns and associated restricted activity reported being hypervigilant of everyday activities that could lead to a fall, negative connotation of older age, the fracture event, pre-fracture abilities and the recovery journey (*Psychological*).

*“Some interviewees described the shock after the injury in words such as ‘everything became a mess’, and that the injury had brought feelings of insecurity and distress. ‘You think, why didn’t I put the light on when I got up [at night]? So, I’m very careful now, almost excessively so.’”*[47]

Across qualitative studies, participants worried about falls and restricted activities outdoors because of poor weather, uneven surfaces, the need to plan in anticipation, and the lack of accessibility in public places and transport. Indoors, participants described higher concerns about falling and activity restriction when activities required climbing stairs or mobilising in the dark. Basic activities of daily living such as toileting were recounted to increase concerns, especially early post discharge (*Environment*).

*“One year later most of the patients had regained their previous level of physical functioning and were able to perform almost all usual activities of everyday life except for some outdoor activities such as taking the bus or biking because they were ‘afraid of falling’.”*[40]

Participants in qualitative studies who expressed more concerns about falling and activity restriction often suggested they did not fully understand or recall information provided. Some felt that healthcare professionals’ suggested activities might lead to a new fall or that they did not fully understand their needs (*Care*)**.**

*“Anna was dependent on help in her home and felt overlooked when busy staff did not recognize her needs: ‘and then all of a sudden they forget a lot of things for example when I used a walker, they could forget to put it where I could reach it and there are a lot of things like that. That’s no good’.”*[46]

Qualitative studies also noted concerns about falling and activity restriction among people living alone, with less involvement in social activities (due to both their own volition and others withdrawal), or with relatives who restricted activities deemed unsafe. This impeded individuals’ ability to challenge themselves and rehabilitate at their own pace[39, 44] (*Social*).

*“The informants had become more bound to their homes and dependent on others to go outside. They saw friends and relatives less often than before and it was up to their relatives to take the initiative.”*[39]

Factors not associated with concerns about falling and activity restriction in observational studies included gender, cognition, positive and negative affect scales, the use of multiple medications and the type of surgery/fracture. Factors with conflicting evidence for an association included pain (*n* = 3 association [50, 52, 54], *n* = 2 no association [48, 49]), age (*n* = 2 association [48, 53], *n* = 2 no association[49, 52]), depression scales (*n* = 1 association [48] *n* = 2 no association [49, 52]), and time since fracture (*n* = 1 association [19], *n* = 2 no association [52, 53]). In qualitative studies, balance and pain have conflicting evidence—with some participants reporting it contributed to activity restriction and others saying it did not (Table 3).

#### Factors to overcome concerns about falling and activity restriction

In observational studies, participants with better balance, strength, and mobility showed less concerns about falling (*Physical*). In qualitative studies, participants who discussed self-determination to remain active and acknowledged that this may ‘look different’ compared to their pre-fracture activity [39, 41, 45–47] – with respect to pacing [39, 41, 45–47] and behavioural adaptations [46] (e.g., only walking over smooth surfaces and for shorter distances)—reported less activity restriction. Many of these participants also celebrated previous challenges overcame or current positive aspects of life [47] (*Psychological*).

*“Being persistent, creative, positive, vigilant, and thoughtful supported a sense of being, and experiences of progress maintained hope and self-confidence. Responsibility was a matter of finding solutions to problems and having duties. Karen had managed baking cookies for my second visit: ‘I tell myself, you HAVE to try, and then when a full baking sheet is ready, I go and sit down for a while. Then back to the oven again. It can take a long time, but I have nothing but time.’* [46]*”*

Qualitative studies pointed out that walking aids and adaptations to the home helped to increase activity and reduce concerns, but required practice and some were considered problematic. Good weather, adequate public transport services and being able to drive, were considered key to increase activity (*Environment*).

*“According to the informants, a walker reduced fear of falling and was necessary at least when walking outside. It was a source of security, but also a nuisance and barrier to moving freely. Another complaint was the inability to use public transportation because the walker was unmanageable in a bus or a car.”*[39]

Participants who reported receiving clear advice on how to safely mobilise and engage with activities without falling, as well as on the consequences of restricted mobility, described less activity avoidance and concerns about future falls. These experiences were augmented through the regular presence of healthcare professionals [45, 46] providing positive feedback and encouragement [44, 46], acknowledging positive progress [44], and listening and acting on participant’s concerns e.g., medications review [47] (*Care*).

*“Balancing risk safely was a consequence of being provided with adequate information. Being informed related to receiving information, feedback, advice or reassurance from healthcare professionals regarding progress. Older people had to rebuild their damaged confidence, unsure of their abilities and needed encouragement to increase their self-efficacy.”*[44]

Qualitative studies described that the presence and company of others provided a sense of connection, motivation, and safety, relieved feelings of loneliness and low mood, which in turn allowed people to move in and outside the house more [45–47]. Others encouragement and awareness of their needed accommodations helped to relieve concerns and activity restriction too. Practically, informal caregivers helped by taking individuals to appointments, shopping, for a walk, or helped to use transport and travel long distances. In a cross-sectional study, however, greater social support was not related to concerns about falling (*Social*).

*“For the interviewees, having a support network, whether formal or informal, was indicated as a way of overcoming dependency to move around and, especially, to walk safely, resulting in better mobility and more active behaviour. Agatha [said]: Yes, just the fact that I had someone, here with me in the afternoon, meant I already felt better. Having human warmth, having company around, I could do more things outside [...] yes, if I had company I’d go by public transport. For example, I’d go to some park, get some fresh air in the park.”* [45]

## Discussion

### Main findings

This mixed-methods systematic review focused on factors related to concerns about falling and activity restriction after hip fracture. We report eight physical, five psychological, five environmental, three social and two care factors contributing to concerns about falling and associated activity restriction after hip fracture. The factors investigated by observational studies were weighted towards the physical, while qualitative studies identified more aspects related to the environment, care, social and psychological. Most factors were reported on by a small number of studies of varying quality.

Findings suggest that concerns about falling and associated activity restriction are more likely to be observed among patients with greater comorbidities; poorer physical and functional abilities post-fracture; less social support; accessibility issues (e.g., living out of area); a lack of, or inability to access, home adaptations; less psychological resources, and/or; poorer perceptions and experiences of the rehabilitation provided at hospital and/or home. Similarly, findings suggest that less concerns and activity restrictions post hip fracture are observed among people with better physical function; higher psychological resilience and positive affect; greater social support; adequate accessibility indoors and outdoors, and; better perceptions and experiences from formal care at the hospital and at home.

### Interpretation

Findings suggest that rehabilitation designed to target physical factors (e.g., strength, function) might help with concerns about falling and activity restriction post-hip fracture. Further reductions in concerns may occur through directly addressing the psychological consequences of a fracture and acknowledging the fact that people will progress at different rates [3], especially in the first three months post-fracture when concerns about falling tend to be high (and potentially reflect an adaptive process) [16, 17]. Our findings suggest that promoting a positive mindset, in addition to building self-confidence and motivation to engage in rehabilitation, may have positive long-term effects on concerns about falling and activity restriction. Indeed, patients have indicated that support and coaching facilitate recovery in daily living after hip fracture [60]. A previous study reported increased physical activity and walking, and reduced concerns about falling, by promoting confidence and motivation for change through motivational interviewing among community-dwelling older adults who had a hip fracture [61].

Few studies examined care factors, despite these being potentially the most amenable to interventions. Where assessed, most focused on communication between healthcare professionals and patients. Effective communication was believed to mitigate concerns about falling and activity restriction by attending medical concerns, increasing access to formal care, providing positive feedback, advice on health consequences, and strategies for safe mobility and ADL engagement [43–47]. Effective communication strategies have been considered crucial by clinicians for engaging patients and improving outcomes after hip fracture [62, 63]. One cross-sectional study of low quality suggested no association between the use of multiple medications and concerns about falling, even though this may reflect frailty and was associated with concerns about falling after hip fracture in a recent study [18]. Further research on potentially modifiable care factors related to concerns about falls and activity restriction after hip fracture is warranted.

The persistence of environmental factors increasing concerns about falling and activity restriction in later stages of recovery aligns with the vast complexities of mobilising as an older adult [64], and the impact of environmental barriers on older adults’ activity and function outdoors [65]. The accessibility of public spaces and services such as transport does not tend to meet the growing demand of people with limited mobility and walking aids [66, 67], especially when additional safety measures are required to avoid a fall [65, 68]. Services that help people go outdoors seem invaluable for those with limited networks to reduce concerns about falling and activity restriction, particularly later post-discharge. However, rehabilitations incorporating outdoor mobility components did not show improvements in falls-related self-efficacy, possibly in part due to an absence of targeting environmental barriers related to mobility [69]. In support, an intervention providing walking maps for the local community environment improved time spent walking outdoors [70].

The current review noted support and company from family, friends, and formal care was associated with a reduction in concerns and increased activity, while relatives' fear and restrictions limited mobility and activity. Findings align with previous reports of patients, informal carers, and clinicians, stating the importance of educating and involving relatives in rehabilitation to improve outcomes across the care continuum [6, 8, 62, 71]. Nevertheless, informal carers report feeling excluded from rehabilitation, struggle to make sense of the information shared, and their relatives needs post-fracture [71]. Findings also emphasise the need and benefit of providing individuals with reduced social networks and who may withdraw from social activities, with alternative means of engagement. Interventions including carers to set goals [72] and to support discharge and home care [73], have showed reductions in concerns about falling at one [73], four [72] and 12 months [74] follow up.

### Future research

Previous evidence suggests concerns about falling at three months was associated with poorer recovery outcomes, an observation that was not seen in the first four weeks [17, 75] or 12 months post-fracture [18]. This suggests that there is a key ‘window’ (between four weeks and three months) to address the fear of falling post-hip fracture, when it appears to reflect a maladaptive process. Further, effective interventions reducing falls concerns have mostly worked for individuals with higher functional ability [72, 73] and were delivered in hospital and community settings [21]. Future interventions should seek to target the factors identified by the current review that predispose an individual to concerns about falling post-fracture. These interventions may employ risk stratification for immutable factors such as pre-fracture function, comorbidities, or stairs at home. Alternatively, they may directly target modifiable factors such as low confidence, social support, or post-fracture function.

### Strengths and limitations

A strength of the review was the use of both observational and qualitative evidence. This provided depth and variety to our findings, expanding on the multiple individual and external factors that impact concerns about falling and activity restriction. We captured studies on varied populations, with qualitative studies tending to focus on under-researched or more vulnerable populations. A main limitation is that we did not draw causal, prospective associations. All studies had a high or moderate risk of bias in study attrition, or analysed data cross sectionally. This issue was also highlighted in a previous review [21]. The quality of the evidence was poor limiting interpretation. For example, no association may reflect a lack of power, a positive/inverse association may occur where authors failed to account for confounding and/or a failure to report on reflexivity. Lastly, there may be different factors for activity restriction and concerns about falling, but we could not clearly differentiate between the two as studies did not report separate results. Our search criteria yielded fewer results than expected so we also hand-searched reference lists [31].

## Conclusion

We observed concerns about falling and activity restriction among individuals following hip fracture with a history of falls, comorbidities, low energy, balance and functionality, who reported low confidence in their own abilities and in rehabilitation. On the contrary, participants with less concerns and activity restrictions had better strength, mobility, social support, formal care experiences, and the ability and confidence to take control over recovery (e.g., adapting behaviours, asking for help). Further, practical social support from informal and formal networks, and the accessibility of indoor and outdoor spaces, seemed essential to overcome fears and increase activity. Findings highlight patient populations who may be at increased risk of longer-lasting concerns and activity restriction resulting in poorer outcomes (e.g., low social support) or who may need more help to overcome worries (e.g., people with anxiety and other comorbidities). Findings also point to an array of potential targets to encourage activity after hip fracture such as self-confidence, strategies for safe mobility and social support from formal and informal networks.

### Supplementary Information

Below is the link to the electronic supplementary material.

Supplementary file1 (PDF 67 KB)

Supplementary file2 (PDF 96 KB)

Supplementary file3 (PDF 71 KB)
