# Supplementary material for: Factors associated with concerns about falling and activity restriction in older adults after hip fracture: a mixed-methods systematic review
Source: Eur Geriatr Med. 2024 Feb 28;15(2):305–32. doi: 10.1007/s41999-024-00936-9 (PMC10997732; doi:10.1007/s41999-024-00936-9)
Supplement: Supplementary file 1 — Supplementary file1 (PDF 67 KB) [file 41999_2024_936_MOESM1_ESM.pdf]

**Appendix I**

<u>Cochrane (Central) – 171 results</u>

**Hip Fracture**

#1 MeSH descriptor: [Hip Fractures] explode all trees
#2 ((hip* or ((femur* or femoral*) near/3 (neck or proximal))) near/4 fracture*):ti,ab,kw
#3 #1 or #2 (2083)

**Fear of falling**

#4 MeSH descriptor: [Accidental Falls] this term only and with qualifiers: [Prevention & control - PC]
#5 MeSH descriptor: [Fear] this term only
#6 (fright* or fear* or afraid):ti,ab,kw
#7 #5 or #6
#8 #4 and #7
#9 (fear* or fright* or afraid) near/5 fall*:ti,ab,kw
#10 "fear of falling":ti,ab,kw
#11 ("Falls Efficacy Scale" or "Mobility Efficacy Scale" or "Survey of Activities and Fear of Falling in the Elderly"):ti,ab,kw
#12 "University of Illinois at Chicago Fear of Falling Measure" or "SAFFE" or "UICFM":ti,ab,kw

#13 exp Sedentary Behavior/ (-")
#14 activit$ adj2 avoidan$:tw

#15 #13 or #14

#16 #8 or #9 or #10 or #11 or #12 or 15

**Prognostic factors**

#17 (Validat* OR Predict*:ti. OR Rule*) OR (Predict* AND (Outcome* OR Risk* OR Model*)) OR ((History OR Variable* OR Criteria OR Scor* OR Characteristic* OR Finding* OR Factor*) AND (Predict* OR Model* OR Decision* OR Identif* OR Prognos*)) OR (Decision* AND (Model* OR Clinical* OR "Logistic Model*")) OR (Prognostic AND (History OR Variable* OR Criteria OR Score* OR Characteristic* OR Finding* OR Factor* OR Model*)) correlat* OR "Stratification*" OR "ROC Curve"[Mesh] OR Discrimina* OR c-statistic OR "c statistic*" OR "Area under the curve" OR "AUC" OR Calibration* OR Indices OR Algorithm* OR Multivariable* Or Correlat* OR Validat* OR Predict* OR Rule OR Predict* AND Outcome* OR Risk* OR Model* OR History OR Variable* OR Criteria OR Scor* OR Characteristic* OR Finding* OR Factor* AND Predict* OR Model* OR Decision* OR Identif* OR Prognos* OR Decision* AND Model* OR Clinical* OR "Logistic Model* OR Prognostic AND History OR Variable* OR Criteria OR Score* OR Characteristic* OR Finding* OR Factor* OR Model* correlat* OR "Stratification* OR "ROC Curve"Mesh OR Discrimina* OR c-statistic OR c statistic* OR Area under the curve OR AUC OR Calibration* OR Indices OR Algorithm* OR Multivariable* Or Correlat*

**Qualitative**

#18 qualitative or interview* or “lived experience*”

#19 MeSH descriptor: [Qualitative Research] or "Qualitative Studies"
#20 #18 or #19

**All**
#30 #20 or #17

#31 #30 and #3 and #16

Medline – 137 articles

**Hip Fracture**

1. exp Femur/
2. Fractures, Bone/ or exp Fracture Fixation/ or Fracture Healing/
3. and/1-2
4. ((hip* or pertrochant* or intertrochant* or trochanteric or subtrochanteric or extracapsular* or ((femur* or femoral*) adj3 (neck or proximal))) adj4 fracture*).tw.
5. exp “Hip Fractures/
6. 4 or 5
7. 6 or 3

**Fear of falling**

8 *Accidental Falls/pc [Prevention & Control]
9. *Fear/
10. (fright$ or fear$ or afraid).tw.
11. 9 or 10
12. 8 and 11
13. ((fear$ or fright$ or afraid) adj5 fall$).tw.
14. "fear of falling".tw.
15. ("Falls Efficacy Scale" or "Mobility Efficacy Scale" or "Survey of Activities and Fear of Falling in the Elderly" or "University of Illinois at Chicago Fear of Falling Measure" or "SAFFE" or "UICFFM" or "Activities Specific Balance Confidence Scale" or "Confidence in Maintaining Balance Scale" or "CON-Fbal").tw.
16. exp “Sedentary Behavior/”
17. "activit$ adj2 avoidan$:tw

18. 16 or 17
19. or/12-15 or 18

**Prognostic factors**

20. (Validat$ OR Predict$.ti. OR Rule$) OR (Predict$ AND (Outcome$ OR Risk$ OR Model$)) OR ((History OR Variable$ OR Criteria OR Scor$ OR Characteristic$ OR Finding$ OR Factor$) AND (Predict$ OR Model$ OR Decision$ OR Identifies OR Prognos$)) OR (Decision$ AND (Model$ OR Clinical$ OR Logistic Models/)) OR (Prognostic AND (History OR Variable$ OR Criteria OR Score$ OR Characteristic$ OR Finding$ OR Factor$ OR Model$)) correlat* OR “Stratification$” OR “ROC Curve”[Mesh] OR Discrimination OR Discriminate$ OR c-statistic OR “c statistic$” OR “Area under the curve” OR “AUC” OR Calibration$ OR Indices OR Algorithm$ OR Multivariable$ Or Correlat$

**Qualitative**

21. qualitative or interview* or “lived experience*”
22. MeSH descriptor: [Qualitative Research] or "Qualitative Studies"
23. 21 or 22

**ALL**

34. 7 and 19 and (23 or 20)

EMBASE 231 articles

**Hip Fracture**

1. exp Femur/
2. Fractures, Bone/ or exp Fracture Fixation/ or Fracture Healing/
3. and/1-2
4. ((hip* or pertrochant* or intertrochant* or trochanteric or subtrochanteric or extracapsular* or ((femur* or femoral*) adj3 (neck or proximal))) adj4 fracture*).tw.
5. exp Hip Fractures/
6. 4 or 5
7. 3 or 6

**Fear of falling**

8 Falling/pc [Prevention]
9 Fear/
10 (fright$ or fear$ or afraid).tw.
11 9 or 10
12 8 and 11
13 ((fear$ or fright$ or afraid) adj5 fall$).tw.
14 "fear of falling".tw.
15 ("Falls Efficacy Scale" or "Mobility Efficacy Scale" or "Survey of Activities and Fear of Falling in the Elderly").tw.
16 ("University of Illinois at Chicago Fear of Falling Measure" or "SAFFE" or "UICFFM").tw.
17 ("Activities Specific Balance Confidence Scale" or "Confidence in Maintaining Balance Scale" or "CON-Fbal").tw.
18. exp Sedentary Behavior/
19. "activit$ avoid$".mp

20. 18 or 19

21. 12 or 13 or 14 or 15 or 16 or 17 or 20

22. 7 and 21

**Prognostic factors**

23 (Validat* or Predict* or Rule* or (Predict* and (Outcome* or Risk* or Model*)) or ((History or Variable* or Criteria or Scor* or Characteristic* or Finding* or Factor*) and (Predict* or Model* or Decision* or Identif* or Prognos*)) or (Decision* and (Model* or Clinical* or Logistic Model)) or (Prognostic and (History or Variable* or Criteria or Score* or Characteristic* or Finding* or Factor* or Model*)) or Correlat* or Stratification* or ROC Curve or Discrimina* or c-statistic* or c statistic* or Area under the curve or AUC or Calibration* or Indices or Algorithm* or Multivariable*).mp.

**Qualitative**

24. qualitative or interview* or ‘lived experience*’ or ‘Qualitative Studies’

25. exp Qualitative Research/
26. 24 or 25
**ALL**

36. 22 and (23 or 26)

**[english only limit)**

CINAHL – 160 studies

**Hip Fracture**

S1 ((MH "Femoral Fractures+") or (MH "Femur/SU") ) or ( femur* or femoral* N3 neck or proximal N4 fracture* ) or ( hip* or pertrochant* or intertrochant* or trochanteric or subtrochanteric or extracapsular* ) or "hip fractures"

**Fear of falling**

S2 (MH "Accidental Falls/PC")
S3 MH Fear
S4 TX (fright$ or fear$ or afraid)
S5 S3 or S4
S6 S2 and S5
S7 TX "fear of falling"
S8 TX ("Falls Efficacy Scale" or "Mobility Efficacy Scale" or "Survey of Activities and Fear of Falling in the Elderly")
S9 TX ("University of Illinois at Chicago Fear of Falling Measure" or "SAFFE" or "UICFFM")
S10 TX ("Activities Specific Balance Confidence Scale" or "Confidence in Maintaining Balance Scale" or "CON-Fbal")
S11 exp "Sedentary Behavior/"
S12 "activit* adj2 avoidan*":ti,ab,kw

S13 S11 or S12
S14 S6 or S7 or S8 or S9 or S10 or S13

**Prognostic factors**

S15 (Validat* OR Predict*:ti. OR Rule*) OR (Predict* AND (Outcome* OR Risk* OR Model*)) OR ((History OR Variable* OR Criteria OR Scor* OR Characteristic* OR Finding* OR Factor*) AND (Predict* OR Model* OR Decision* OR Identif* OR Prognos*)) OR (Decision* AND (Model* OR Clinical* OR "Logistic Model*")) OR (Prognostic AND (History OR Variable* OR Criteria OR Score* OR Characteristic* OR Finding* OR Factor* OR Model*)) correlat* OR "Stratification*" OR "ROC Curve"[Mesh] OR Discrimina* OR c-statistic OR "c statistic*" OR "Area under the curve" OR "AUC" OR Calibration* OR Indices OR Algorithm* OR Multivariable* Or Correlat*

**Qualitative**

S16 (MH "Qualitative Studies") or (qualitative or interview* or "lived experience*" or "Qualitative research")

**ALL**

S17 S1 and S14 and (S15 or S16)

PsychInfo - 37 articles

**Hip Fracture**

1. Hips/
2. fracture*.tw.
3. ((hip* or pertrochant* or intertrochant* or trochanteric or subtrochanteric or extracapsular* or ((femur* or femoral*) adj3 (neck or proximal))) adj4 (fracture* or injur*)).tw.
4. (1 and 2) or 3
5. Hip Fracture$.mp.
6. 4 or 5

**Fear of falling**

7. "fear of falling".mp. [mp=title, abstract, heading word, drug trade name, original title, device manufacturer, drug manufacturer, device trade name, keyword heading word, floating subheading word, candidate term word]
8. ("Falls Efficacy Scale" or "Mobility Efficacy Scale" or "Survey of Activities and Fear of Falling in the Elderly" or "University of Illinois at Chicago Fear of Falling Measure" or "SAFFE" or "UICFFM" or "Activities Specific Balance Confidence Scale" or "Confidence in Maintaining Balance Scale" or "CONFbal").mp. [mp=title, abstract, heading word, drug trade name, original title, device manufacturer, drug manufacturer, device trade name, keyword heading word, floating subheading word, candidate term word]
9. 7 or 8
10. exp Sedentary Behavior/
11. "activit* avoidan*".mp
12. 10 or 11
13. 9 or 12

**Prognostic factors**

14. (Validat* or Predict* or Rule* or (Predict* and (Outcome* or Risk* or Model*)) or ((History or Variable* or Criteria or Scor* or Characteristic* or Finding* or Factor*) and (Predict* or Model* or Decision* or Identif* or Prognos*)) or (Decision* and (Model* or Clinical* or Logistic Model)) or (Prognostic and (History or Variable* or Criteria or Score* or Characteristic* or Finding* or Factor* or Model*)) or Correlat* or Stratification* or ROC Curve or Discrimina* or c-statistic* or c statistic* or Area under the curve or AUC or Calibration* or Indices or Algorithm* or Multivariable*).mp. [mp=title, abstract, heading word, drug trade name, original title, device manufacturer, drug manufacturer, device trade name, keyword heading word, floating subheading word, candidate term word]

**Qualitative**

15. (qualitative or interview or 'lived experience' or 'Qualitative Research' or 'Qualitative Studies').mp. [mp=title, abstract, heading word, drug trade name, original title, device manufacturer, drug manufacturer, device trade name, keyword heading word, floating subheading word, candidate term word]

**All**

26. 6 and 13 and (13 or 14)

PEDro – 11 articles

**Hip Fracture + RCT**

Abstract & title: fracture*
Body part: thigh or hip
Method: clinical trial

**Fear of falling= 21**

Abstract & title: fear
Body part: thigh or hip
Method: clinical trial
**Fracture* thigh or hip= 309**

References used:

**Hip Fracture**

Crotty M, Unroe K, Cameron ID, Miller M, Ramirez G, Couzner L. Rehabilitation interventions for improving physical and psychosocial functioning after hip fracture in older people. Cochrane Database of Systematic Reviews 2010, Issue 1. Art. No.: CD007624. DOI: 10.1002/14651858.CD007624.pub3. Accessed 10 June 2022.

Handoll HHG, Cameron ID, Mak JCS, Panagoda CE, Finnegan TP. Multidisciplinary rehabilitation for older people with hip fractures. Cochrane Database of Systematic Reviews 2021, Issue 11. Art. No.: CD007125. DOI: 10.1002/14651858.CD007125.pub3. Accessed 10 June 2022.

**Fear of falling**

Kendrick D, Kumar A, Carpenter H, Zijlstra GAR, Skelton DA, Cook JR, Stevens Z, Belcher CM, Haworth D, Gawler SJ, Gage H, Masud T, Bowling A, Pearl M, Morris RW, Iliffe S, Delbaere K. Exercise for reducing fear of falling in older people living in the community. Cochrane Database of Systematic Reviews 2014, Issue 11. Art. No.: CD009848. DOI: 10.1002/14651858.CD009848.pub2. Accessed 10 June 2022.

**Prognostic factors**

Geersing, G.J., Bouwmeester, W., Zuithoff, P., Spijker, R., Leeflang, M. and Moons, K., 2012. Search filters for finding prognostic and diagnostic prediction studies in Medline to enhance systematic reviews. *PloS one*, *7*(2), p.e32844.

**Qualitative**

Shaw, R.L., Booth, A., Sutton, A.J., Miller, T., Smith, J.A., Young, B., Jones, D.R. and Dixon-Woods, M., 2004. Finding qualitative research: an evaluation of search strategies. *BMC medical research methodology*, *4*(1), pp.1-5.
