# Supplementary material for: Factors associated with concerns about falling and activity restriction in older adults after hip fracture: a mixed-methods systematic review
Source: Eur Geriatr Med. 2024 Feb 28;15(2):305–32. doi: 10.1007/s41999-024-00936-9 (PMC10997732; doi:10.1007/s41999-024-00936-9)
Supplement: Supplementary file 2 — Supplementary file2 (PDF 96 KB) [file 41999_2024_936_MOESM2_ESM.pdf]

Appendix II

Table 1. Characteristics of Observational Studies

Author, year, country	Study design	Setting (year)	Inclusion criteria	Sample size (all numbers)	Participants characteristics
Benzinger, 2010, Germany	Observational prospective study	single geriatric rehabilitation (2007-2009)	aged 65 years or older admitted after surgical treatment of a proximal femoral fracture, living in the community at the time of fracture and near the site<br><br>Exclusion - medical problems not related to hip fracture but heavily influencing general health, severe cognitive impairment, insufficient understanding of the German language, not available for screening, lack of testing capacity, admitted on Fridays or before holidays	88 screened<br><br>15 excluded and 14 drop-outs<br><br>59 completed follow up<br><br>51 analysed	Mean age (SD)= 82.9 (7.5)<br><br>Women n (%) = 45 (88)<br><br>Nursing home: community= 9:42
Bower, 2019, USA	Observational prospective study	eight hospitals in the St. Louis area (2008-2012)	Within 1 week of hip fracture, age 60 years or older and had a primary diagnosis of hip fracture to be surgically repaired.<br><br>Exclusion - major depression prior to fracture, metastatic cancer, and dementia or severe cognitive impairment that did not improve by hospital discharge	501 enrolled from previous study<br><br>263 completed and analysed in current study	Mean age (SD)= 77.54 (8.78)<br><br>Women n (%) = 198 (75.3)<br><br>Living at home with no assistance pre-fracture: 81%
Goto, 2020, Japan	Observational prospective study	Nagasaki Memorial Hospital in Nagasaki,	Patients older than 65 years who were admitted to the emergency unit with a femoral neck fracture or femoral	242 screened identified<br><br>170 excluded	*Light group (n=50)*<br><br>Mean age (SD)= 82.8 (6.5)<br><br>Women n (%) = 44 (88)

		Japan (2014-2018)	trochanteric fracture after a fall diagnosed by radiography.<br><br>Exclusion - the transfer from another hospital, acute exacerbation, repeat fracture, discharge within 4 weeks after surgery, inability to walk within 4 weeks after surgery, and decline in cognitive function	72 enrolled and analysed	*Severe group (n=22)*<br><br>Mean age (SD)= 82.9 (7.2)<br><br>Women n (%) = 20 (90)
Jellesmark, 2012, Denmark	Sequential explanatory mixed-method study, cross-sectional for quantitative data	Two orthopedic department s at Copenhage n University Hospital (2010)	Hip fracture, age 65 years and older, community-dwelling, ability to walk independently at discharge, and MMSE score within the normal range, i.e. score 24–30<br><br>For qualitative subset - high degree of FEAR OF FALLING, varying levels of functional ability and ability to verbalize the situation in detail<br><br>Exclusion - readmission to hospital for a week or more, inability to read or understand Danish and living outside the Copenhagen area.	204 patients screened<br><br>71 eligible<br><br>33 enrolled and analysed	Women: men = 26:7<br><br>Median age (range) = 81 (65-92)<br><br>Living alone n(%)= 24 (73%)<br><br>3> comorbidities n(%)= 13 (40%)
Kulmala, 2007, Finland	Retrospective observational study	Recruitmen t setting unclear	60- to 85-year-old patients operated at the local hospital for collum or trochanter fracture within 6 months to 7 years prior to the baseline measurements.<br><br>Exclusion – those with neurological diseases (including memory disorders), progressive severe	452 informed about study<br><br>132 interviewed to ensure inclusion and exclusion criteria were met	*Women* N=54,<br><br>Mean age (SD) [women] =76 (6.2)<br><br>*Men* N=25,<br><br>Mean age (sd)= 73.6 (7.4)

			illnesses, or inability to walk outdoors without other person's assistance	79 eligible, enrolled, and analysed	
Nagai, 2014, Japan	Cross-sectional study	Community-dwelling outpatients (year not reported)	Inclusion- THA<br><br>Exclusion- Patients with neurological impairment (e.g.stroke, Parkinson'sdisease, paresis of the lower limbs), cardio vascular disease, arthrorheumatism, visual impediment, dizziness, severe cognitive impairment or hiposteoarthritis without THA	381 invited<br><br>359 consented (94% response rate)<br><br>145 excluded<br><br>214 analysed	Mean age (SD)=64.2 (NA)<br><br>Women = 100%
Portegijis, 2012, Finland	Cross-sectional study, analyses of pretrial data of 2 RCTs of physical rehabilitation	Patient records at the Central Finland Central Hospital (2004–2005 and 2008–2010)	Community-dwelling people older than 60 years who had been operated on for femoral neck or trochanteric fracture (6 weeks to 7.5 years after a fall-related hip fracture).<br><br>Exclusion- inability to move outdoors without assistance from another person, amputation of a lower limb, severe progressive or neurologic diseases, alcoholism, and severe memory problems (Mini-Mental State Examination score).	149 eligible<br><br>19 excluded<br><br>130 analysed	Mean age (SD)= 77.6 (7.2)<br><br>Women (%) = 98 (75.4)
Salpakoski, 2010, Finland	cross sectional study, part of a larger RCT	Central Hospital of Central Finland (2004-2005)	Community-dwelling men and women aged 60–85, operated for a femoral neck or trochanteric fracture during the past 8 months to 7.5 years.<br><br>Exclusion- inability to move outdoors without assistance from another person, amputation of a lower limb, severe progressive or neurological diseases, and severe memory	452 invited<br><br>132 interviewed<br><br>100 invited<br><br>78 enrolled and analysed	*Physical inactivity group*<br><br>Mean age (SD)= 75 (7.5),<br><br>Women n (%) = NA (73)<br><br>*Physical activity*<br><br>Mean age (SD)= 75.3 (6.4)<br><br>Women n (%) = NA (65)

			problems (diagnosed dementia or MMSE).		
Tu, 2021, Australia	Observational prospective study	Rehabilitation centres in Victoria, Australia (June 2011 and October 2012)	Patients who have sustained a hip fracture following a fall, were aged 60 years or over, and were preparing for discharge from the rehabilitation centre to community living (either in their own home or a family home).<br><br>Exclusion- low English proficiency, a cognitive impairment recorded in their medical record or low level of pre-fracture mobility (unable to walk more than 10 m indoors independently with or without a gait aid).	192 screened<br>75 eligible<br>44 enrolled<br>34 completed follow up	Mean age (SD): 82 (8)<br>Women n (%) = 21 (62)
Whitehead 2003, Australia	Observational prospective study	Flinders Medical Centre (between August 1998 and June 1999)	Patients over the age of 60 years with a hip fracture, community dwelling and assessed as being cognitively intact (MMSE > 23)	214 identified<br>183 consented<br>64 excluded<br>119 analysed	Mean age (SD)= 81.3 (6.2) years<br>Women n (%) = 51 (69.9)

Mini-Mental State Examination (MMSE). Randomised controlled trial (RCT)

Table 2. Characteristics of qualitative studies

Author, year, country	Study design	Setting (Year)	Inclusion/ Exclusion criteria	Sample size (all numbers)	Participants characteristics	Aim
Abrahamsen, 2022, Denmark	Longitudinal interview study, semi-structured interviews	Orthogeriatric ward in Denmark (2018)	Purposive sampling for different gender, marital status and pre-fracture mobility.	12 interviewed at the first time point	Mean age (range)= 85.3 (65-103)<br>Women: men= 10:2 (83.3% women)	To explore the impact of a hip fracture on elderly patients' everyday life from

			Inclusion - Hip fracture patients undergoing surgery, 65 years or older. Able to understand and speak Danish.	6 (50%) interviewed on two time points<br>4 (33%) interviewed on four time points	Married or cohabitating = 5/12 (41.6%).<br>Returned to home after hip fracture= 11/12 (91.6%)	their perspective and at different time points
Gesar 2017, Sweden	Explorative inductive, Semi-structured interview	Three Swedish hospitals (2013-2014)	Inclusion - patients after hip fracture surgery, live an independent life before the<br>fracture, aged 65 years or older, were previously healthy (none or mild systemic disease), had no cognitive impairment and able to speak and understand Swedish.	30 participants (from 1st study, Gesar 2017a)<br>25 agreed to participate	17 were aged 80 years and older.<br>Women: men ratio= 22:3 (88% women)<br>Moved into a nursing home = 3/25	To reveal how previously healthy people, aged 65 years and older, described how they had adapted to daily life four months after a hip fracture
Griffiths, 2015, UK	Interview study	Major trauma centre in the West Midlands of the UK (2012)	Purposefully sampled cohort participants who had reached 4 weeks or 4 months following their hip fracture and had consented to be approached for interview.	31 participants, 41 interviews:<br>21 interviewed on one occasion	Mean age (SD, range)= 81.5 years (SD 9.2, 61–96).<br>Women: men = 20:11 (64.5% women)	To explore with patients and, where appropriate, their carers, what they consider to be important outcomes and to explore variation across this patient group

Author, year, country	Study design	Setting (Year)	Inclusion/ Exclusion criteria	Sample size (all numbers)	Participants characteristics	Aim
			Inclusion - participants from an existing cohort study, the Warwick Hip Trauma Evaluation, that started in January 2012. This is a cohort of all patients admitted with a hip fracture to a single major trauma centre in the West Midlands of the UK.	10 interviewed twice;<br><br>19 interviews with patient only<br><br>14 with carer only<br><br>8 with the patient/carer dyads.		
Jellesmark, 2012, Denmark	Sequential explanatory mixed method, in-depth interviews	Two orthopedic departments in Denmark, 3 months post hospital discharge (March to May 2010)	Inclusion - hip fracture, age 65 years and older, community-dwelling, ability to walk independently at discharge, and mini-mental state examination (MMSE) score within the normal range, i.e. score 24–30<br><br>For qualitative subset - high degree of FEAR OF FALLING, varying levels of functional ability and ability to	33 included in<br><br>the study<br><br>19 had a high degree of fear of falling<br><br>4 purposely selected	Women: men= 3:1 (75% women)<br><br>Kate, a 91-year-old woman, was the only informant who needed ADL-assistance prior to the hip fracture due to visual and auditory impairment. She became more dependent after the fracture.	To assess self-reported FEAR OF FALLING and functional ability among community-dwelling elderly people 3–6 months post hospital discharge after a hip fracture.<br><br>To investigate the association between FEAR OF FALLING and functional ability.<br><br>And to explore the lived experience of FEAR OF FALLING and disability

**Author, year, country**	**Study design**	**Setting (Year)**	**Inclusion/ Exclusion criteria**	**Sample size (all numbers)**	**Participants characteristics**	**Aim**
			verbalize the situation in detail		Hans, a 92-year-old man became dependent on assistance and the walker after the fracture.<br><br>Kirsten, a 74-year-old woman became confined to her home after the fracture<br><br>Kim, an 82-year-old woman was still independent and went outside her house every day	when recovering from a hip fracture.
McMillan, 2012, Scotland	Grounded theory	Two local health authorities which incorporated three<br><br>acute hospitals<br><br>(December 2007-April 2009)	Inclusion- sustained a fall-induced hip fracture, had been discharged home within the previous 3 months and were willing and able to provide informed consent.<br><br>Purposive sampling was employed initially, then theoretical	19 included before theoretical saturation	Mean age (range)= 79 (67-89) years<br><br>Women: men= 15:4 (79% women)<br><br>Lived alone= 10<br><br>Lived with family or partner = 9	To explore the post discharge concerns of older people after fall-induced hip fracture to increase understanding and awareness of issues that may impact on recovery and rehabilitation.

**Author, year, country**	**Study design**	**Setting (Year)**	**Inclusion/ Exclusion criteria**	**Sample size (all numbers)**	**Participants characteristics**	**Aim**
			sampling aimed to source those with particular experiences that would develop the emerging theory.			
McMillan, 2013, Scotland	Grounded theory	District general hospitals taking part in the Scottish Hip Fracture Audit (2007 - 2009)	Inclusion- sustained a fall-induced hip fracture, had been discharged home within the previous 3 months and were willing and able to provide informed consent.<br><br>Purposive sampling was employed initially, then theoretical sampling aimed to source those with particular experiences that would develop the emerging theory.	19 included	Mean age (range)= 79 (67-89) years<br><br>Women: men= 15:4 (79% women)<br><br>Lived alone= 10<br><br>Lived with family or partner = 9	To explore the post discharge concerns of older people after fall-induced hip fracture. This paper reports specifically on balancing risk, which was a key strategy that older people employed in their efforts to take control.
Moraes, 2020, Brazil	Semi-structured interview, phenomenological approach	Older adults who agreed to participate in a RCT	Convenience and theoretical saturation sampling	29 eligible<br><br>11 included	Women: men =8:3 (72% women)<br><br>Lived alone = 4 (36%)<br><br>Age of participants not reported	To understand the experience of older adults after hip fracture surgery due to a fall regarding barriers and facilitators

Author, year, country	Study design	Setting (Year)	Inclusion/ Exclusion criteria	Sample size (all numbers)	Participants characteristics	Aim
		Data were collected over a period of four months (March to June 2017)	Men and women aged 60 years old or older, eligible for a randomized, 12-month follow-up trial aimed at assessing the effectiveness of a physical exercise program to improve functional mobility post-hip fracture in older adults in the late rehabilitation phase.			related to sedentary behaviour
Rasmussen, 2018, Denmark	Phenomenological-hermeneutic	Non-university hospitals (year not stated).	Inclusion: 65 years or older, with pre-hip fracture limitation of functional ability, not living in a nursing home, no other actual fractures than hip, and able to speak about experiences in Danish.	13 included<br><br>11 interviewed at follow up	Mean age (range)= 74.5 (73-92)<br>Women: men= 11:2 (85% women)<br>3 married, 10 widowers.	To explore facilitators for and<br>barriers to being active experienced by older people<br>during the first 6 months after hip fracture surgery.
Taylor, 2010, Australia	Phenomenological theoretical framework and grounded theory methods, in-depth semi-structured interviews	patients receiving either inpatient or outpatient rehabilitation after hip fracture, (recruitement	Purposive sampling - Individuals who could provide information about community ambulation after hip fracture were deliberately selected.	24<br><br>12 living at home and receiving	Inpatient<br>Inpatient rehabilitation after hip fracture, an average of 3.9 weeks [standard deviation (SD): 2.4] after surgery.	To explore mobility levels around the home and in the community before and after hip fracture.

Author, year, country	Study design	Setting (Year)	Inclusion/ Exclusion criteria	Sample size (all numbers)	Participants characteristics	Aim
		site was not stated)	Inclusion - receiving rehabilitation after a fracture of the neck of femur; aged at least 60 years, living independently in the community (not in supported care) before the fracture if an inpatient or currently living independently in the community if an outpatient; have a standardised mini-mental state Examination score greater than 24, and be able to converse in English.	outpatient rehabilitation<br>+<br>12 while receiving inpatient rehabilitation	Mean age (SD)= 76.0 (5.7)<br>Women: men= 8:4 (67% women)<br>Lives alone: 4 (33%)<br>Mean comorbidities (SD)= 4.5 (2.2)<br>Outpatient<br>Outpatient rehabilitation services an average of 12.2 weeks (SD: 4.9) after hip fracture.<br>Mean age (SD)= 80.4 (7.2)<br>Women: men = 9:3 (82% women)<br>Lives alone= 1 (8%)<br>Mean comorbidities (SD)= 5.8 (2.1)	
Ziden, 2010, Sweden	Phenomenological, interview study	At Sahlgrenska University Hospital in Gothenburg.	Purposeful selection of<br>individuals with different backgrounds (marital status, living	102 participants in previous RCT	Age range= 66–93,<br>Women: men= 13:2 (87% women)	To explore experienced long-term consequences of a hip fracture and conceptions of what influences hip fracture

Author, year, country	Study design	Setting (Year)	Inclusion/ Exclusion criteria	Sample size (all numbers)	Participants characteristics	Aim
		People who had previously participated in RCT	conditions, age, pre-fracture<br><br>functional status, type of fracture and participation in<br><br>rehabilitation programmes)<br><br>Inclusion for previous RCT- 65 years or older, community-living at the time of injury, with no life-threatening disease or severe cognitive impairment and able to understand and speak Swedish.	15 interviewed	5 married, 10 widower/widow.	recovery among community-living elderly people 1 year after discharge.

American Society of Anesthesiologists (ASA). Randomised controlled study (RCT)
