# Supplementary material for: Factors associated with concerns about falling and activity restriction in older adults after hip fracture: a mixed-methods systematic review
Source: Eur Geriatr Med. 2024 Feb 28;15(2):305–32. doi: 10.1007/s41999-024-00936-9 (PMC10997732; doi:10.1007/s41999-024-00936-9)
Supplement: Supplementary file 3 — Supplementary file3 (PDF 71 KB) [file 41999_2024_936_MOESM3_ESM.pdf]

Appendix III

Table 1. Quality appraisal of observational studies using the quality in prognosis studies (QUIPS) tool.

Author, year	Study Participation	Study Attrition	Prognostic Factor Measurement	Outcome Measurement	Confounding	Statistical analysis and reporting
Benzinger, 2010	Low	Moderate	Moderate	Moderate	High	Moderate
Bower, 2020	Moderate	High	Moderate	High	Moderate	Low
Goto, 2020	Low	Moderate	Moderate	Low	High	Moderate
Jellesmark, 2012+	Low	N/A*	Moderate	Low	High	Moderate
Kulmala, 2007	Low	Low	Moderate	Low	Low	Low
Nagai, 2014	Moderate	NA*	Moderate	Moderate	Moderate	High
Portegijis, 2012	Low	NA*	Moderate	Moderate	Moderate	Low
Salpakoski, 2010	Low	NA*	Low	Low	Moderate	Low
Tu, 2021	Low	Moderate	Low	Low	Low	Low
Whitehead, 2003	Low	High	Moderate	Low	High	Moderate

*Cross-sectional studies
+From mixed-method study

Table 2. Quality appraisal of qualitative studies using the critical appraisal skills programme (CASP) checklist.

Author, year	Q1	Q2	Q3	Q4	Q5	Q6	Q7	Q8	Q9	Q10
Abrahamsen 2022	Y	Y	U	U	Y	U	Y	Y	U	U
Gesar 2017	Y	Y	U	Y	Y	N	Y	Y	U	U
Griffiths 2015	Y	Y	Y	Y	Y	Y	Y	Y	Y	Y
McMillan 2012	Y	Y	Y	Y	Y	U	Y	Y	Y	Y
McMillan 2013	Y	Y	U	Y	Y	N	Y	Y	Y	Y
Moraes 2020	Y	Y	Y	Y	Y	U	U	U	Y	Y
Jellesmark 2012+	Y	Y	U	Y	N	N	U	Y	U	Y
Taylor 2010	Y	Y	Y	Y	Y	Y	Y	Y	Y	Y
Rasmussen 2018	Y	Y	Y	Y	Y	Y	Y	Y	Y	Y
Ziden 2010	Y	Y	Y	Y	Y	Y	Y	Y	Y	Y

+From mixed-method study

Y:Yes, N:No, U: Uncertain
Q1: Was there a clear statement of the aims of the research?
Q2: Is a qualitative methodology appropriate?
Q3: Was the research design appropriate to address the aims of the research?
Q4: Was the recruitment strategy appropriate to the aims of the research?
Q5: Was the data collected in a way that addressed the research issue?
Q6: Has the relationship between researcher and participants been adequately considered?
Q7: Have ethical issues been taken into consideration?
Q8: Was the data analysis sufficiently rigorous?
Q9: Is there a clear statement of findings?
Q10: How valuable is the research?
